# Expression patterns of serum MicroRNAs related to endothelial dysfunction in patients with subclinical hypothyroidism

**DOI:** 10.3389/fendo.2022.981622

**Published:** 2022-09-06

**Authors:** Xuelin Yao, Ying Wang, Li Wang, Mingfeng Cao, Aifang Chen, Xinhuan Zhang

**Affiliations:** ^1^ Shandong Academy of Medical Sciences, Shandong First Medical University, Taian, China; ^2^ Department of Endocrinology, the Second Affiliated Hospital of Shandong First Medical University, Taian, China; ^3^ Department of Pharmacy, the Second Affiliated Hospital of Shandong First Medical University, Taian, China; ^4^ Ultrasound Department, the Second Affiliated Hospital of Shandong First Medical University, Taian, China

**Keywords:** subclinical hypothyroidism, atherosclerosis, endothelial dysfunction, circulating microRNAs, differentially expressed miRNAs

## Abstract

**Background:**

Increasing evidence has shown that elevated Thyroid stimulating hormone (TSH) levels are positively correlated with atherosclerosis (ATH) in patients with subclinical hypothyroidism (SCH). Some researchers found that the dysfunction of Endothelial Cells (ECs) in SCH plays an important role in the pathogenesis of ATH in SCH, but the association remains controversial.

**Objectives:**

To determine the expression profiles of serum microRNAs critical to the function of Endothelial cells (ECs) may help reanalyze the possible mechanism underlying ATH in SCH and the association between ATH and SCH.

**Methods:**

We used qRT-PCR to perform microRNA profiling and analysis in normal control subjects (NC), patients with SCH alone (SCH), patients with SCH and ATH (SCH+ATH), and patients with ATH without SCH (ATH).

**Results:**

Both miR-221-3p and miR-222-3p showed a decreasing expression trend between the SCH and SCH+ATH groups. In addition, miR-126-3p and miR-150-5p showed a stepwise decrease from the NC to SCH groups and then to the SCH+ATH or ATH group. miR-21-5p was unregulated in the SCH, SCH+ATH, and ATH groups. Furthermore, elevated levels of miR-21-5p in SCH+ATH group were higher than SCH and ATH group. No differences were found in the levels of miR-150, miR-126, miR-221 and miR-222 between the ATH and the SCH+ATH subjects.

**Conclusions:**

miR-21-5p may be involved in the atherosclerosis process in patients with SCH (SCH and SCH+ATH groups). miR-150-5p may be sensitive risk markers for predicting endothelial dysfunction in patients with ATH (ATH and SCH+ATH groups).

## Introduction

Subclinical hypothyroidism (SCH) is an endocrine and metabolic disorder that is characterized by elevated serum thyroid-stimulating hormone (TSH) above the upper limit of reference with normal serum free thyroid hormones (FT4 and FT3) (1). Recently, several studies have shown that SCH is associated with hypercoagulability (2), cardiovascular disease (3), dyslipidaemia (4), and increased carotid artery atherosclerosis (5). Atherosclerosis is a chronic inflammatory response with a complex pathological mechanism. Endothelial cell (EC) dysfunction, autophagy dysfunction and microRNAs are involved in the development of atherosclerosis (6, 7). Dysfunction of EC is the most important manifestation of the earliest pathophysiological process of atherosclerosis. Several pathological processes were involved in the atherosclerotic process, including the selective recruitment of circulating monocytes from the blood into the intima, the internalization of modified lipoproteins into foam cells, the production and aggregation of multiple chemokines and growth factors, result in the generation of atherosclerotic plaques (8–10). In addition, some researchers have found TSH receptor expression in microvascular ECs (11) and vascular smooth muscle cells (12). Elevated TSH levels are the key initiation factor of atherosclerosis, directly induce oxidative stress and damage EC function (13). Therefore, the association between SCH and EC dysfunction in SCH needs to be fully elucidated from different perspectives.

MicroRNAs (miRNAs) are a class of endogenous, small noncoding RNAs that are 21~23 nucleotides in length (14). They are involved in the regulation of gene expression at the post-transcriptional level by degrading their target miRNAs and/or by inhibiting translation, thus controlling many different processes and pathways within the cell (15). miRNAs have been reported to process a wide range of many biological processes, such as cell proliferation (16), angiogenesis (17), stress response (18), glucose and lipid metabolism (19), tumor development (20) and other physiological processes. However, the pathogenetic mechanisms of endothelial dysfunction-specific circulating miRNAs in patients with SCH have not been elucidated thoroughly.

Our study aimed to evaluate the expression profiles of serum miRNAs critical to the function of ECs in patients with SCH or ATH, and reanalyze the association between atherosclerosis and SCH from a new perspective. Serum miRNAs include miR-21 (21), miR-150 (22), miR-126 (23), miR-221 (24), miR-222 (25), and miR-210 (26), which have been confirmed to be related to EC dysfunction and were selected into our study. miR-126 is an important regulator of EC biology, including angiogenesis, vascular repair, inflammation activation and apoptosis (27). MiR-21 plays an important role in the regulation of cardiovascular diseases, including changes in the functions of ECs, vascular smooth muscle cells and macrophage-foam cells (28). miR-210 can modulate the angiogenesis of ECs upon hypoxic condition *via* vascular endothelial growth factor (VEGF) signaling (29), miR-221/-222 can inhibit the migration, proliferation and angiogenesis of ECs (25), miR-150 can promote EC proliferation and plays an important role in angiogenesis and proliferation (30).

## Materials and methods

### Research subjects

**Figure 1:**
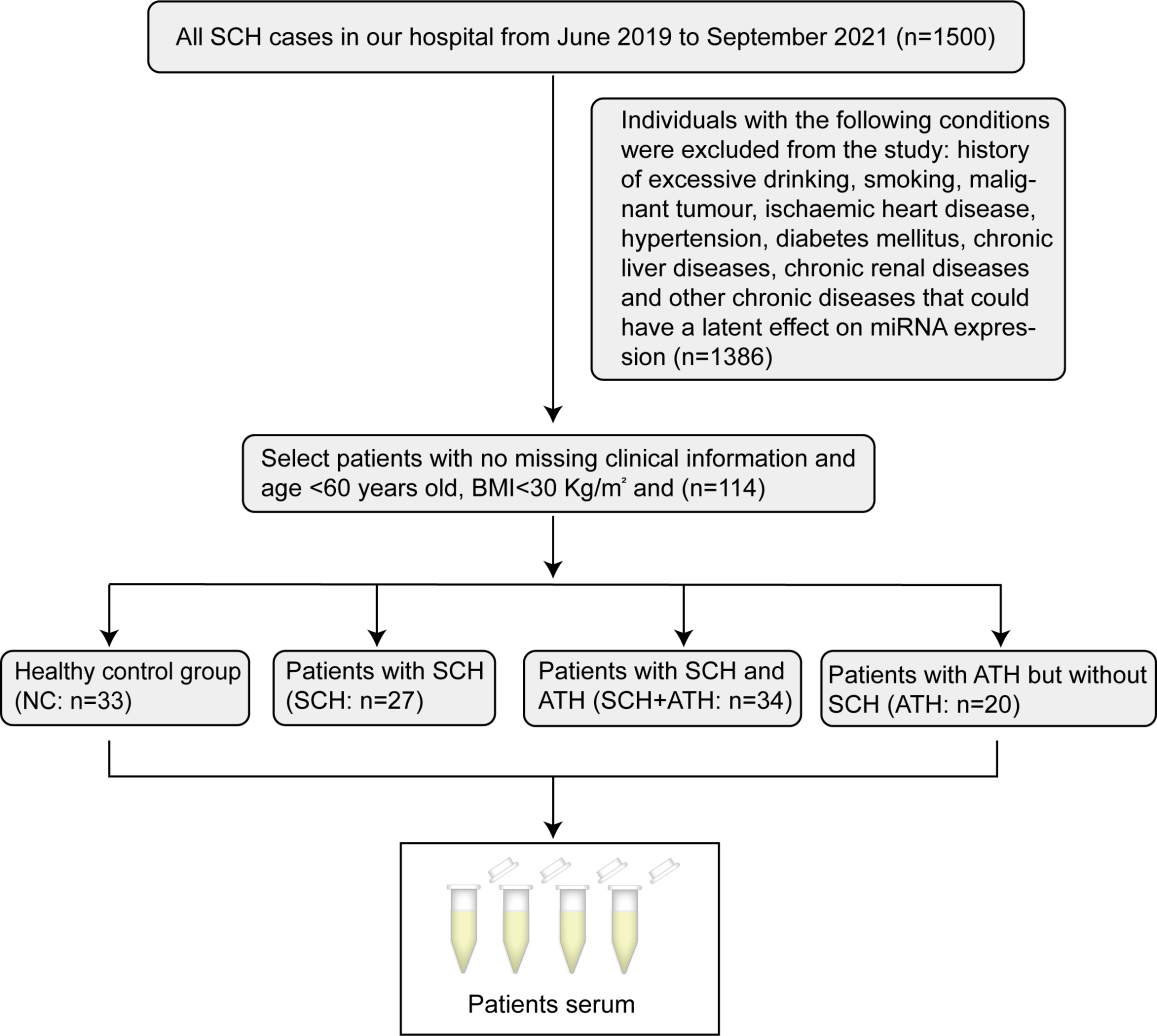
Flow chart in the study protocol.

The diagnoses of SCH (1), atherosclerosis (ATH) (31), lipid metabolism abnormalities (32), and flow-mediated dilatation (FMD) (33) were made in accordance with the guidelines. Carotid artery measurement methods and carotid intimal thickening standards refer to the diagnostic consensus on carotid intimal thickening and plaque formation (31) and the 2018 Chinese Guidelines for Prevention and Treatment of Hypertension (34). Atherosclerosis (ATH) risk by detecting atheroma plaques and quantifying intima-media thickness (IMT) of the common carotid artery. Diagnosis of early atherosclerosis according to the max-IMT (define normal when max-IMT<0.9mm and arotid intima-media thickness when max-IMT is between 0.9 and 1.3mm, carotid intima-media atheromatous plaque formation when max-IMT >1.3mm). All subjects recruited in our study were divided into four groups (**Figure 1**): healthy control group (NC: n = 33), patients with SCH (SCH: n = 27), patients with SCH and ATH (SCH + ATH: n = 34), and patients with ATH but without SCH (ATH: n = 20). We screened approximately 1500 newly diagnosed patients with SCH in the outpatient Department of Endocrinology from the Second Affiliated Hospital of Shandong First Medical University from January 2019 to September 2021. Moreover, to avoid the influence of confounding factors, individuals with the following conditions were excluded from the study: history of excessive drinking, smoking, malignant tumor, ischaemic heart disease, hypertension, diabetes mellitus, chronic liver diseases, chronic renal diseases and other chronic diseases that could have a latent effect on miRNA expression. All subjects were asked to complete a self-report questionnaire and provided an overnight fasting blood sample. The study was performed in accordance with the Declaration of Helsinki and was approved by the Ethics Committee of the Second Affiliated Hospital of Shandong First Medical University. Written informed consent was obtained from all subjects in advance. This study was registered in the Clinical Trial Registry (Effects of L-thyroxine Replacement on Serum Lipid and Atherosclerosis in Hypothyroidism, NCT01848171).

### Measurement of anthropometric and biochemical parameters

According to our previous study (35), all baseline characteristics, such as height (to the nearest 0.1 cm) and weight (to the nearest 0.1 kg), were recorded, and BMI was calculated as weight (kg) divided by height squared (m^2^). Blood pressure was measured in the right arm using a standard mercury sphygmomanometer with an appropriately sized cuff while participants were in a seated position. Blood was drawn with minimal trauma from an antecubital vein in the morning after a 12 h overnight fast and then stored at -80°C in aliquots, partly for blood biochemistry analysis and partly for miRNA extraction. Serum TSH, FT3, FT4, nitric oxide (NO), total cholesterol (TC), triglycerides (TG), low-density lipoprotein (LDL-c) and high-density lipoprotein (HDL-c) were measured in accordance with the kit instructions. To reduce the interassay differences, all measurements were performed at the same clinical laboratory of the Second Affiliated Hospital of Shandong First Medical University.

### Assessment of carotid intima-media thickness and blood flow-mediated dilation

According to Zhang’s previous research (35), all patients underwent a carotid intima-media thickness **(**CIMT) examination of the bilateral carotid arteries using a color ultrasonic diagnostic (Toshiba Aplio 500 Ultrasound Scanner). Subjects rested in the supine position with the head titled backwards while the study was performed in a temperature-controlled room (25 ℃). The bilateral carotid arteries, internal carotid artery (ICA), common carotid artery (CCA) and carotid bulb (sinus) were longitudinally scanned at different angles, and the thickest part of the vessel wall and plaques along the artery wall were explored. CIMT is defined as the visible distance between the haemal junction and the foreign membrane junction. Three measurements were performed near the thickest point of the IMT and at two adjacent points (1 cm upstream and 1 cm downstream). The mean values of the three measurements are represented as the IMT. The maximum values of the CCA, ICA and sinus of carotid artery were regarded as the maximum (max) IMT. The mean intima thickness at the six left and right points was the mean carotid intima thickness (36). Carotid artery measurement methods and carotid intimal thickening standards refer to the diagnostic consensus on carotid intimal thickening and plaque formation (31) and the 2018 Chinese Guidelines for Prevention and Treatment of Hypertension (34): max CIMT ≤ 0.9 mm is normal, max CIMT > 0.9 mm is considered intimal thickening, and max CIMT ≥ 1.3 mm indicates atherosclerotic plaque formation.

According to Corretti’s study (33), FMD levels were measured by a vascular endothelial function tester produced by UNEX, Japan (UNEXEF18G). The patients were asked to stop using vascular drugs for 24 h and avoid tobacco use for 4-6 h. In a quiet temperature-controlled room with an empty stomach and calm state, the patient laid on his/her back and fully exposed the right arm. First, five heartbeats with a basic inner diameter of the brachial artery were recorded at the end of diastole, the baseline diameter was measured, and then the sphygmomanometer cuff was tied to the upper arm of this side. The lower edge of the cuff was located 2-3 cm above the elbow stripes. The pressure was approximately 50 mmHg (1 mmHg = 0.133 kPa) so that the blood flow was blocked. After 5 min, the cuff was deflated, the diameter of hyperemia was measured and recorded within 60-90 s after deflation. The change in arterial diameter after occlusion in response to hyperemia over the baseline diameter was calculated according to the equation below. Each participant was investigated by the same physician, who was blind to the patients’ clinical data and risk factors.

Equation: %FMD is defined as: $\;\frac{\left(HyperemiaDiameter-BaselineDiameter\right)}{BaselineDiameter}\times 100\%$

### Total microRNA isolation

According to our previous study (35) and other studies (37, 38), a synthetic *C. elegans* miR-39 miRNA mimic (cel-miR-39: catalogue no. 219610) is suitable for the normalization of circulating miRNA preparation. It was introduced as a spike-in control to monitor technical variations in RNA recovery, and it was used as the normalization control to monitor the effectiveness of RT-PCR and qPCR. First, total RNA containing small RNAs was isolated from 200 μL serum using the miRNeasy Serum/Plasma Kit (Qiagen: catalogue no. 217184), and 3.5 μL of the miRNeasy Serum/Plasma Spike-In Control (1.6 ×10^8^ copies/μL working solution) was then added to the plasma samples to extract again (recommended by Qiagen) according to the manufacturer’s instructions. After the addition of a denaturing solution, 3.5 μL of the miRNeasy Serum/Plasma Spike-In Control (1.6 ×10^8^ copies/μL working solution) was added to the plasma samples to extract again (recommended by Qiagen). Eventually, total RNA containing miRNAs was eluted in 14 μL of RNase-free water. A NanoDrop 1000 (NanoDrop, Wilmington, DE, USA) was applied to quantify the concentration of RNA, reverse transcribed to cDNA and quantitative real-time PCR for specific miRNAs was performed.

### Reverse transcription to cDNA and quantitative real-time PCR for specific miRNAs

According to our previous study (35), the miScript II RT Kit (Qiagen, catalogue no. 218160) was used to reverse 50 ng of serum RNA containing small RNAs transcribed to cDNA. Reverse transcription reactions contained a total of 50 ng of RNA, 5 μL 5x miScript HiSpec Buffer, 2.5 μL 10x miScript Nucleics Mix, 2.5 μL miScript Reverse Transcriptase Mix, and RNase-free water up to 25 μL. The reactions were incubated at 37°C for 60 min, followed by inactivation of the reaction by incubating at 95°C for 5 min.

Specific miRNA quantification was performed by SYBR Green-based real-time PCR using a miScript SYBR Green PCR Kit (Qiagen, catalogue no. 218073). Detailed information about the six candidates is presented in **Supplemental Table 1** and can be found online at http://www.mirbase.org/. Each 20 μL PCR mix contained 10 μL 2x QuantiTect SYBR Green PCR Master Mix, 2 μL 10x miScript Universal Primer, 2 μL 10x QuantiTect miScript Primer Assay, 2.5 μL RNase-free water, and 3.5 μL cDNA as template. The reactions were incubated at 95°C for 15 min, followed by 55 cycles of 94°C for 15 s and 55°C for 30 s and 70°C for 30 s. All reactions were run in duplicate. The threshold cycle (Ct) was calculated using the second-derivative max method. Ct values greater than 40 were treated as undetermined (38). The relative expression of each miRNA after normalization to cel-miR-39 is displayed as 2^- (Ct [miRNA] -Ct [cel-miR-39])^.

## Statistics

Data are presented as the mean ± SEM or raw numbers. The normality of the variables was assessed by the Shapiro-Wilk test. Data from the Kruskal-Wallis test. Normal distribution was analyzed by one-way analysis of variance (ANOVA) and the LSD *post hoc* multiple comparisons test. The Mann-Whitney U test was performed to compare data that were not normally distributed. The chi-square test was employed to compare gender distributions. Logistic regression analysis was used to detect the risk factors of atherosclerosis in patients with SCH. Spearman’s correlations were performed to explore the associations between TSH, lipid parameters, miRNAs and CIMT, NO, and FMD. GraphPad Prism 8.0.1 (GraphPad Software, Inc., La Jolla, CA) was used to perform an agglomerative hierarchical cluster analysis based on the miRNA expression patterns. Receiver operating characteristic (ROC) curve analysis was performed to assess the prognostic ability of candidate miRNAs for arteriosclerosis. All data were processed by the SPSS software package for Windows version 25.0 (SPSS, lnc, Chicago, USA). All statistical tests were two-sided, and a value of *p<* 0.05 was defined as statistically significant.

## Results

### Significant differences were shown in the following indicators among the four groups: lipidaemia, level of TSH and CIMT, FMD, and NO

**Figure 2:**
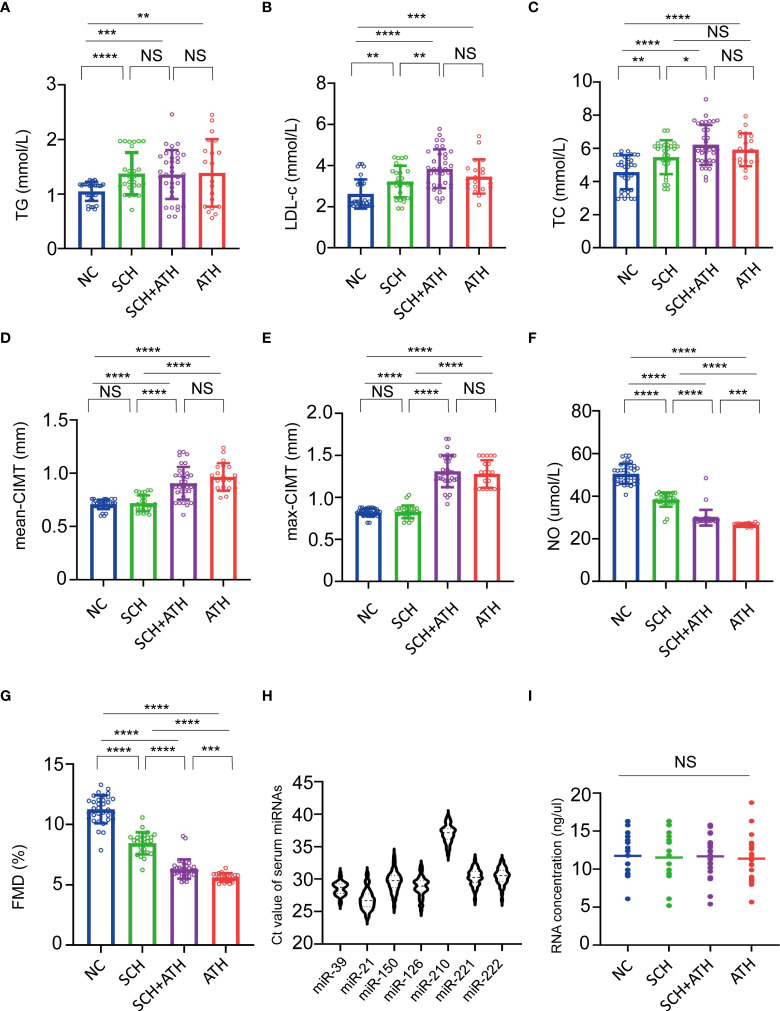
**(A–G)**. Multiple comparisons of baseline characteristics among NC, SCH, SCH+ATH and ATH subjects. *P< 0.05, **P< 0.01, ***P< 0.001, ****P< 0.0001, NS means no significance. **(H)** The six miRNAs and cel-miR-39 served as the spike-in control all showed reliable threshold cycle (Ct) values in all subjects. The horizontal lines indicate the mean. **(I)** The total RNA concentration among the four group were discovered no differences significantly. NS means no significance.

As displayed in **Figures 2A–G**, serum TC and LDL-c showed significant stepwise increases from the NC to SCH group and then to the SCH+ATH or ATH group. Serum TG was significantly higher in all patients with SCH (SCH and SCH+ATH) as compared to that in the control group, whereas no difference was found between the SCH and SCH+ATH group. In addition, no significant differences were found between the (SCH+ATH and ATH groups) and (SCH+ATH and SCH groups). Both NO and FMD showed significant decreases from the NC group to the SCH group and then to the SCH+ ATH and ATH group. Although the mean IMT and max IMT showed significant stepwise increases from the SCH group to the SCH+ATH group, no significant differences were identified between the (NC and SCH groups) and (SCH+ATH and ATH groups). In addition, no significant differences were found in other clinical factors, including DBP, gender, BMI, FT3, FT4 and HDL-c (**Table 1**).

**Table 1:** Charactices of Subjects inthis study.

	NC	SCH	SCH+ATH	ATH	*p * value
Gender (M/F)	33 (9/24)	27 (6/21)	34 (3/31)	20 (5/15)	0.25
Age (y)	44.91 ± 7.828	42.22 ± 11.623	45.50 ± 10.900	52.05 ± 6.684	0.008
BMl (kg/m2)	24.05 ± 3.503	24.73 ± 2.426	25.03 ± 3.153	26.28 ± 2.743	0.084
SBP (mmHg)	118.36 ± 8.306	ll5.19 ± 7.028	120.09 ± 12.878	124.85 ± 5.842	0.007
DBP (mmHg)	76.76 ± 4.101	75.74 ± 10.021	79.91 ± 9.395	76.60 ± 7.877	0.194
TC (mmol/L)	4.57 ± 1.030	5.47 ± 1.021	6.21 ± 1.207	5.92 ± 0.989	<0.001
TG (mmol/L)	1.05 ± 0.175	1.37 ± 0.387	1.36 ± 0.449	1.39 ± 0.619	0.004
LDL-c (mmol/L)	2.62 ± 0.710	3.23 ± 0.771	3.85 ± 0.944	3.47 ± 0.833	<0.001
HDL-c (mmol/L)	1.26 ± 0.236'	1.21 ± 0.248	1.26 ± 0.305	1.40 ± 0.339	0.155
FT3 (mmol/L)	4.83 ± 0.652	4.44 ± 0.721	4.26 ± 1. ll6	4.91 ± 0.949	0.015
FT4 (mmol/L)	14.21 ± 1.657	14.65 ± 1.884	14.35 ± 2.221	14.60 ± 2.237	0.822
TSH (mlU/mL)	3.68 ± 0.426	7.25 ± 2.393	9.02 ± 3.797	3.47 ± 0.901	NA
mean-IMT (mm)	0.71 ± 0.046	0.72 ± 0.075	0.91 ± 0.026	0.97 ± 0.130	NA
max-IMT (mm)	0.82 ± 0.046	0.83 ± 0.075	1.31 ± 0.189	1.28 ± 0.164	NA
NO (umol/L)	53.17 ± 4.301	39.10 ± 1.780	29.02 ± 0.529	26.72 ± 0.671	<0.001
FMD (%)	11.68 ± 1.031	8.26 ± 0.740	6.13 ± 0.437	5.61 ± 0.353	<0.001

NA, not applicable. p<0.01 was defined significant.

In the qPCR analysis, all six miRNAs and the spike-in control miRNA mimic (cel-miR-39) showed reliable Ct values in all subjects (**Figures 2H–I**). All six candidate miRNAs showed no differences among the four groups. miR-210 expression was the least abundant, with a median Ct of 37.17, whereas miR-21-5p expression was the most abundant, with a median Ct of 26.69.

### Different expression profiles of the six selected miRNAs among the four groups

**Figure 3:**
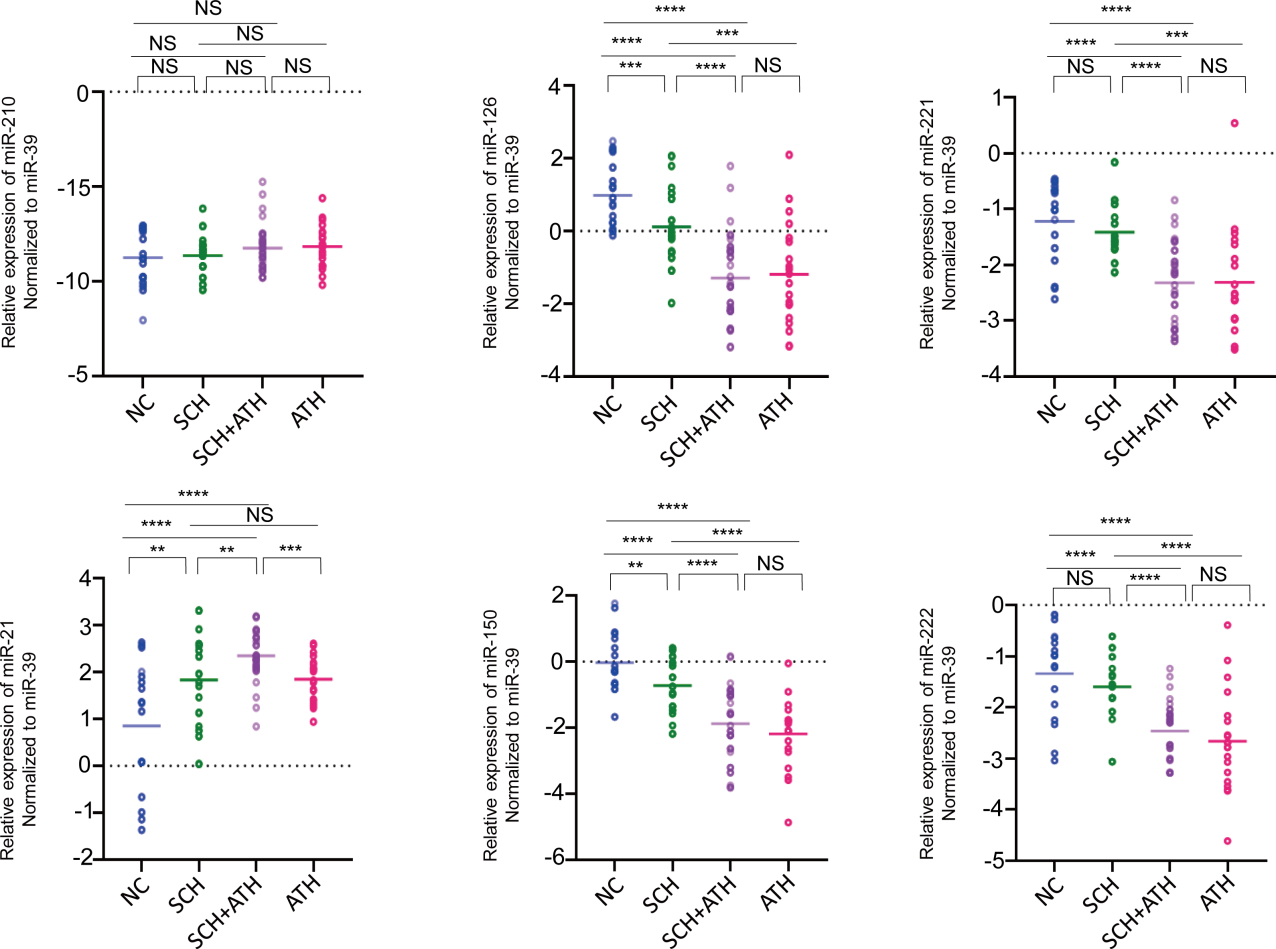
The relative expression levels of 6 candidate serum miRNAs (shown in log2 scale) among all subjects. Cel-miR-39 is a synthetic C. elegans miR-39 miRNA mimic. The horizontal lines indicate the mean. The dotted lines indicate the zero. P value was generated by one-way ANOVA test followed by the LSD post hoc multiple comparisons test. *P< 0.05, **P< 0.01, ***P< 0.001, ****P< 0.0001, NS means no significance.

As described in **Figure 3**, the relative expressions of miR-21-5p in the ATH group (1.847 ± 0.480), SCH group (1.830 ± 0.961) and the SCH+ATH group (2.349 ± 0.523) was significantly higher than that in the NC group (0.850 ± 1.348), however, there was no significant difference between the SCH and ATH groups (*P* = 0.945). The miR-210 critical to hypoxia-induced vascular proliferation showed a slight but insignificant stepwise trend from the NC group (-8.746 ± 1.368) to the SCH group (-8.649 ± 1.041) and then to the SCH+ATH group (-8.239 ± 1.195), the ATH group (-8.156 ± 1.159). Both miR-126-3p and miR-150 showed a significant decreasing trend from the NC group (0.986 ± 0.857 and -0.030 ± 0.860, respectively) to the SCH group (0.115 ± 0.989 and -0.726 ± 0.814, respectively) and then to the SCH+ATH group (-1.292 ± 1.196 and -1.879 ± 1.096, respectively), whereas no differences were identified between the SCH+ATH and the ATH groups. Both miR-221-3p and miR-222-3p were significantly downregulated in the SCH+ATH (1.220 ± 0.732 and -1.414 ± 0.506, respectively), SCH groups (-1.339 ± 0.879 and -0.726 ± 0.814, respectively), and ATH groups (-2.312 ± 0.928 and -2.659 ± 0.980, respectively), but there was no significant difference between the SCH+ATH and ATH groups (*P* = 0.977 and *P* = 0.347, respectively).

### Correlation between the levels of TSH, blood lipids, CIMT, NO, FMD and objective microRNAs

As shown in **Table 2**, Spearman’s correlation analysis indicated that TC and LDL-c were positively correlated with TSH, TG and CIMT (including mean CMT and max CIMT) and negatively correlated with NO and FMD. TG was only negatively correlated with NO and FMD, however, no correlation between TSH and CIMT (mean CIMT and max CIMT) was observed.

**Table 2:** Spearman’s Correlation Analysis of some parameters and Expression of Six MicroRNAs in All Subjects.

Parameter		TSH	TC	TG	LDL-c	Mean-CIMT	Max-CIMT	FMD	NO
TC	R	0.275	1.000	0.394	0.734	0.365	0.413	-0.454	-0.407
	*p*	0.003	NA	<0.001	<0.001	<0.001	<0.001	<0.001	0.001
LDL-c	R	0.329	0.734	0.494	1.000	0.454	0.468	-0.388	-0.334
	*p*	<0.001	<0.001	<0.001	NA	<0.001	<0.001	<0.001	<0.001
TG	R	0.115	0.394	1.000	0.494	0.175	0.187	-0.220	-0.200
	*p*	0.223	<0.001	NA	<0.001	0.062	0.046	0.019	0.033
Mean-CIMT	R	0.046	0.365	0.175	0.454	1.000	0.813	-0.680	-0.660
	*p*	0.624	<0.001	0.062	<0.001	NA	<0.001	<0.001	<0.001
Max-CIMT	R	0.198	0.413	0.187	0.468	0.813	1.000	-0.757	-0.712
	*p*	0.035	<0.001	0.046	<0.001	<0.001	NA	<0.001	<0.001
log2miR-21exp	R	0.396	-0.037	-0.050	0.005	0.206	0.205	-0.327	-0.338
	*p*	<0.001	0.696	0.594	0.958	0.028	0.028	<0.001	<0.001
log2miR-150exp	R	-0.260	-0.341	-0.226	-0.358	-0.505	-0.576	0.667	0.635
	*p*	0.005	<0.001	0.016	<0.001	<0.001	<0.001	<0.001	<0.001
log2miR-126exp	R	-0.305	-0.372	-0.285	-0.399	-0.486	-0.567	0.651	0.610
	*p*	<0.001	<0.001	0.002	<0.001	<0.001	<0.001	<0.001	<0.001
log2miR-210exp	R	0.033	0.130	0.132	0.149	0.121	0.225	-0.146	-0.168
	*p*	0.730	0.167	0.163	0.114	0.200	0.016	0.121	0.074
log2miR-221exp	R	-0.154	-0.381	-0.161	-0.349	-0.578	-0.547	0.568	0.523
	*p*	0.103	<0.001	0.086	<0.001	<0.001	<0.001	<0.001	<0.001
log2miR-222exp	R	-0.124	-0.335	-0.159	-0.317	-0.560	-0.559	0.562	0.523
	*P*	0.190	<0.001	0.091	0.001	<0.001	<0.001	<0.001	<0.001
FMD	*R*	-0.220	-0.454	-0.220	-0.388	-0.680	-0.757	1.000	0.948
	*P*	0.019	<0.001	0.019	<0.001	<0.001	<0.001	NA	<0.001
NO	*R*	-0.177	-0.407	-0.200	-0.334	-0.660	-0.660	0.948	1.000
	*P*	0.060	<0.001	0.033	<0.001	<0.001	<0.001	<0.001	NA

Correlations were presented as related coefficient (R) and significance (p), p < 0.05 was considered as significant.

R means correlation coefficient, p < 0.05 was considered as significant. NA, not applicable.

With regard to the six selected miRNAs in our research (**Table 2**), miR-21-5p was positively correlated with TSH and CIMT and negatively correlated with NO and FMD, but no correlations were demonstrated between miR-21-5p and blood lipids (TC, TG and LDL-c) as well as CIMT (mean CIMT and max CIMT). All the other downregulated miRNAs, including miR-150-5p, miR-126-3p, miR-221-3p and miR-222-3p, were negatively correlated with TSH, blood lipids profiles (TC and LDL-c, besides TG), and CIMT as well as positively correlated with NO and FMD. No correlation between miR-210 and blood lipids (TC, TG and LDL-c), TSH, CIMT, NO, FMD was observed.

### The independent risk factors for atherosclerosis in patients with SCH were analyzed by logistic regression analysis

**Figure 4:**
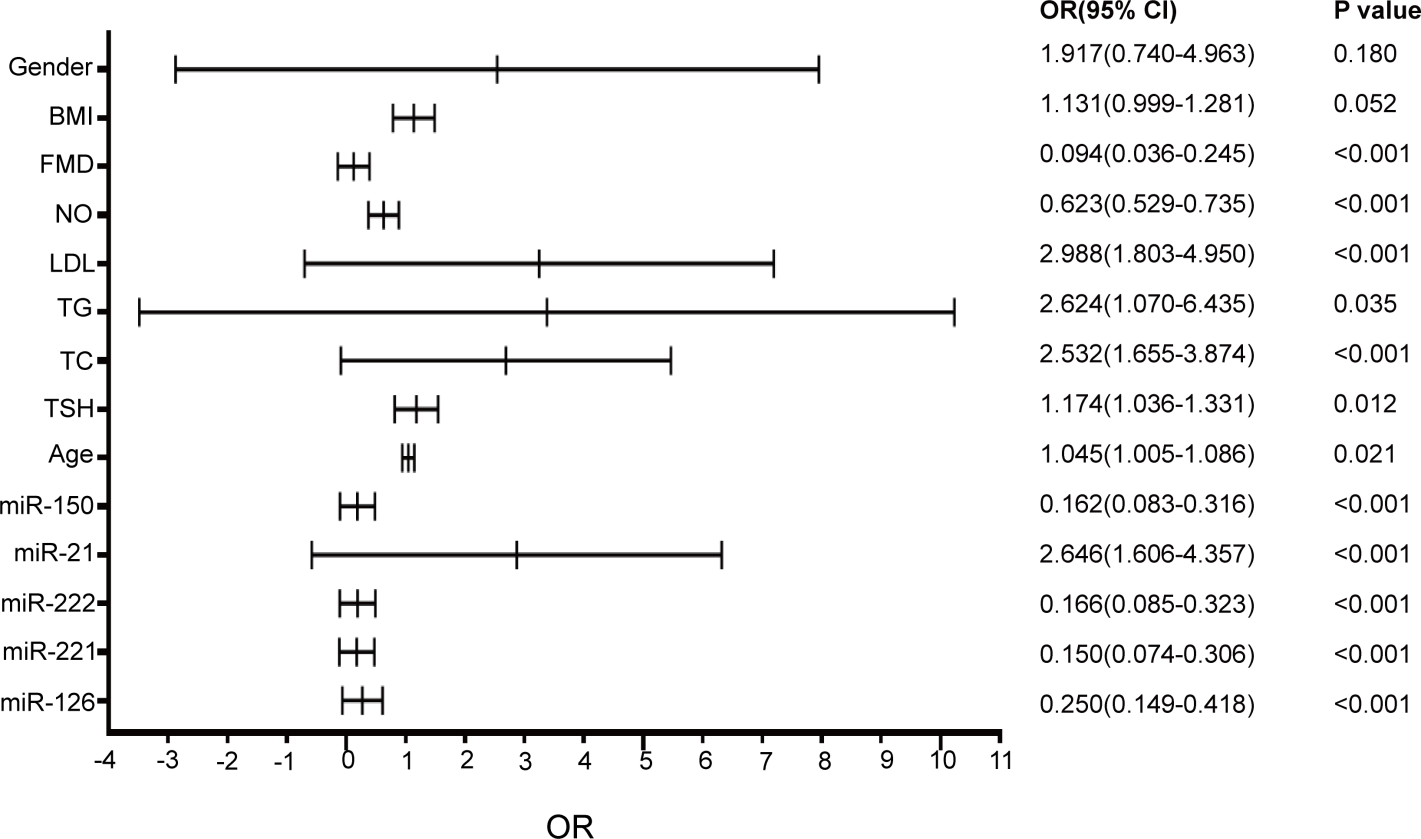
The variable parameters of affecting atherosclerosis. The horizontal lines indicate the OR (Odds Ratio). The dotted lines indicate the variable parameter value. P< 0.05 was defined significant. OR, Odds Ratio; 95% CI, 95% confidence interval.

As shown in **Figure 4**, TC, LDL-c, FMD, NO, miR-221, miR-222, miR-21, miR-150 and miR-126 were associated with ATH. miR-21 (OR = 2.646, *P<*0.001), LDL-c (OR = 2.988, *P<*0.001) and TC (OR = 2.532, *P<*0.001) were positively correlated with ATH. Other parameters including FMD, NO, miR-126, miR-150, miR-221 and miR-222 were negatively correlated with ATH. However, BMI, age, TSH, gender and TG were not found to be significantly correlated with pathological progression of ATH.

### Differential expression of five serum target miRNAs was analyzed by hierarchical agglomerative clustering analysis gathering most individuals among the four groups

**Figure 5:**
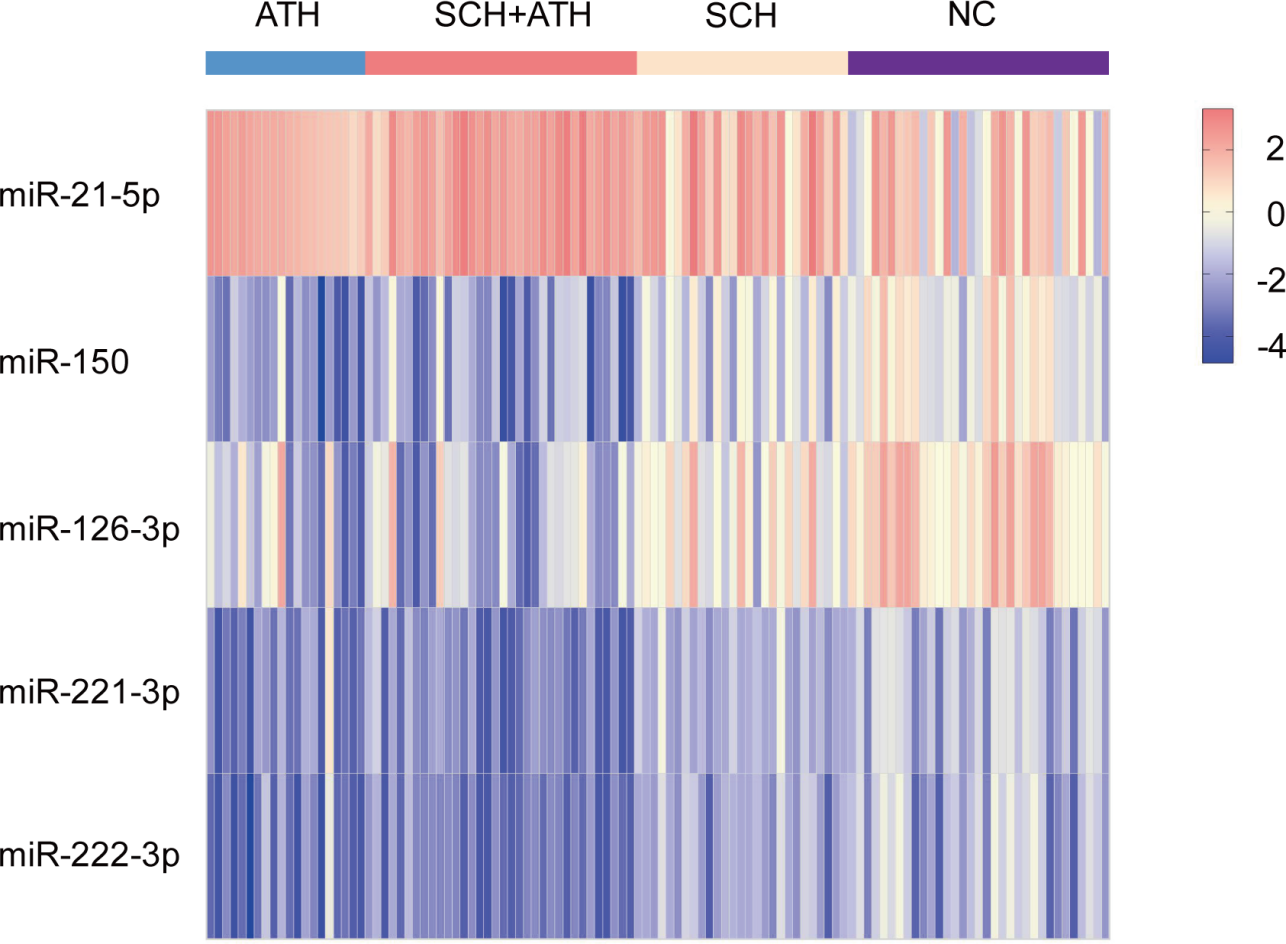
The different expression of five miRNAs could be agglomerated in hierarchical agglomerative clustering analysis.

Hierarchical clustering analysis can find the linear combinations of miRNAs that maximize the probability of correctly assigning subjects (39). The expression levels of the five miRNAs were significantly different among the four groups and were used to generate a heat map diagram. Hierarchical clustering analysis based on the five differentially expressed serum miRNAs could separate a majority of subjects from each group (**Figure 5**). Heatmap showing the relatively specific expression patterns of the five differentially expressed serum miRNAs among the four groups.

### ROC curves of six candidate serum miRNAs for evaluation of all atherosclerosis patients and all subclinical hypothyroidism patients

**Figure 6:**
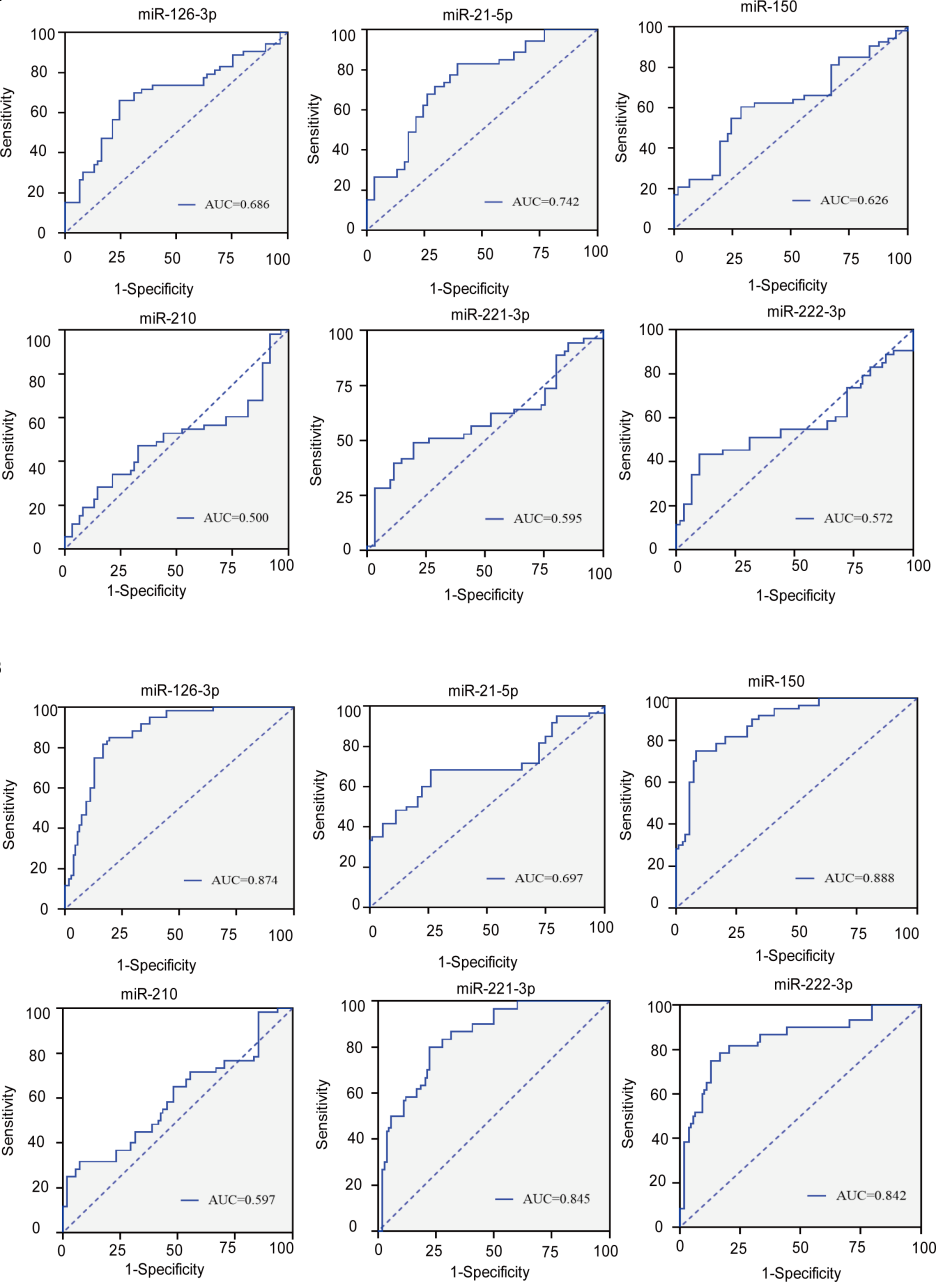
**(A)** Roc curves for six circulating miRNAs to distinguish the all SCH patients (SCH+ATH and SCH) from the (NC and ATH) subject. **(B)** Roc curves for six circulating miRNAs to distinguish the all ATH patients (SCH+ATH and ATH) from the (NC and SCH) subject. AUC, area under curve; CI, confidence interval.

Receiver operating characteristic (ROC) curves were constructed to evaluate the sensitivity and specificity of six candidate serum miRNAs in predicting endothelial dysfunction of all subclinical hypothyroidism patients and atherosclerosis patients. The areas under the ROC curves (AUCs) of the six miRNAs are displayed in **Figure 5**. miR-21-5p (**Figure 6A**) had the largest AUC: 0.742 (95% confidence interval 0.652-0.833) among the six candidate miRNAs to differentiate the all-subclinical hypothyroidism patients including SCH+ATH and SCH patients. miR-150 (**Figure 6B**) had the largest AUC: 0.888 (95% confidence interval 0.828-0.947) among the six candidate to differentiate for detection of all atherosclerosis patients including the SCH+ATH and ATH groups from all cases.

## Discussion

In this study, we reanalyzed the association between blood lipid profiles, TSH, CIMT and endothelial dysfunction, including increased NO production and FMD using high-resolution B-mode ultrasound in patients with SCH. Results in our analysis showed that patients in the SCH, SCH+ATH, and ATH groups differed from NC subjects in the mean value of TG, TC, LDL-c, CIMT, FMD and NO. Furthermore, TSH was positively correlated with TC and LDL-c, and was negatively correlated with FMD and NO, suggesting that TSH may be conducive to dyslipidemia, arteriosclerosis and EC injury. This finding is in line with the results of other studies that have proven these associations (40). With regard to the correlations between increased lipids levels including TC and LDL-c, and CIMT, and FMD, NO, blood lipids were all positively correlated with mean-CIMT and max-CIMT, as well as negatively correlated with FMD and NO as expected. Previous studies have associated NO, FMD and CIMT with vascular endothelial dysfunction and demonstrated that increased TSH may lead to a reduction in endothelium-derived NO production (41, 42). These data indicate that hyperlipidemia may have a more direct influence on endothelial dysfunction than TSH does, which may be due not only to the extent but also to the duration of the TSH elevation associated with ATH. TSH may play a physiological role in promoting ATH mainly through lipid dysregulation, as shown by several but not all previous studies (13).

miRNAs, as key regulators in gene expression networks, can influence many biological processes (14). Increasing evidence has suggested that circulating miRNAs in the blood showed promise as biomarkers for various physiopathological conditions, including cancer, neurodegeneration, diabetes and other diseases (43). Evidence from the CECSID trial has shown that miR22-3p downregulation ameliorates visceral adiposity (44) and miR122-5p overexpression affects the extracellular matrix through MMP-2 modulation in Type 2 Diabetes patients (45). Detailed validation of the preanalytical steps affecting miRNA detection and quantification is critical when considering the use of individual miRNAs as clinical biomarkers (46). Our qRT-PCR analysis confirmed the results of previous reports that circulating miRNAs can be reliably measured in serum from humans (47). Five of the six atherosclerosis-specific miRNAs had significantly different expression levels among the four groups, and their levels were correlated with TSH, lipid parameters, NO, FMD and CIMT. This result suggests that miRNAs related to endothelial dysfunction may be involved in the process of ATH in patients with SCH. In addition, the dysregulation of miRNAs (including miRNA-21, miRNA-31, miRNA-221, and miRNA-222) was also validated by RT-PCR analysis in an independent set of FFPE tissues in papillary thyroid carcinoma, these results highlight the applicability of miRNA expression patterns as potential markers of human cancer (48).

Logistic regression analysis showed that FMD, NO, miR-150, miR-221, miR-222 and miR-126 were negatively correlated with ATH in SCH patients. TC, LDL-c and miR-21 were positively correlated with ATH. Lipid metabolism disorders play a significant role in the pathological progression of ATH, which may be because hyperlipidemia can produce numerous lipid oxides deposited in the vascular endothelium, affected the activation of the coagulation and fibrinolysis systems. resulted in the dysfunction of ECs and accelerating the occurrence of ATH. Patients with elevated serum NO levels and FMD have a significant lower risk of ATH and EC dysfunction. NO is produced in almost all tissues and organs, exerting a variety of biological actions (49). Many key steps in the atherogenic process are inhibited by NO/platelet adhesion and aggregation, adhesion molecule and chemokine expression, inflammatory cell infiltration as well as smooth muscle cell migration and proliferation (50). Previous studies have shown that serum NO levels are easier to detect and more clearly reflect vascular endothelial dysfunction (51), which serves as an early marker of atherosclerosis (52).

As expected, miR-126-3p was significantly downregulated in patients with thickened CIMT and negatively correlated with TSH, blood lipids and CIMT, with the strongest positive correlations with FMD (R = 0.651, *P<* 0.001) and NO (R = 0.610, *P<* 0.001), suggesting that miR-126-3p may be involved in the critical targets and pathways of pro-ATH and endothelial dysfunction in patients with SCH. miR-126 is a miRNA that is well characterized to regulate vascular angiogenesis, vascular repair, inflammation activation and apoptosis (53). For instance, miR-126 may affect EC proliferation and permeability by targeting sprouty related EVH1 domain 1 (SPRED1) and phosphatidylinositol 3 kinase regulatory subunit 2 (PIK3R2), and positively regulate angiogenesis (54). miR-126-3p protected vascular ECs from hypoxia/reoxygenation (H/R)-induced injury and inflammation and inhibits hydrogen peroxide-induced EC apoptosis (55). Another miRNA associated with hypoxia is miR-210 (29). Overexpression of miR-210 directly downregulated Ephrin-A3 and enhanced the capillary-like formation and chemotaxis induced by VEGF, which contributed to the regulation of the angiogenesis response to ischaemia (56). Our data suggested that the expression level of miR-210 in the circulation was generally low, and no significant difference was found between miR-210 and TSH, CIMT, FMD, NO and lipid levels among the four groups. A plausible explanation for this inconsistency may be that carotid artery ATH only indicates the early stage of ATH; at this point, critical regulators related to hypoxia have not changed dramatically.

miR-150, an endothelial-specific miRNA, promotes EC proliferation, vascular angiogenesis and apoptosis (30). Our results showed that miR-150 had a stepwise decrease in the NC group to the SCH group and then to the SCH+ATH and ATH group, was negatively correlated with TSH, TC, LDL-c and CIMT, and was positively correlated with NO and FMD. These data indicated that downregulation of miR-150 is related to dyslipidemia and elevated TSH and CIMT, which increase the risk of endothelial injury and arteriosclerosis. In addition, ROC curves showed that miR-150 was the most sensitive and suitable risk biomarker for all ATH patients than any other miRNA in our research. In this study, miR-150 was mainly considered between dyslipidemia and ATH, which was consistent with many previous studies (30, 57). We speculate that the down-regulation of miR-150 expression may be critical to the vascular endothelial dysfunction and even pro-ATH in SCH patients, and tightly closed to lipid metabolism. Next, we will investigate the specific expression patterns of miR-150 at different TSH levels and try to explore the possible targets and pathways.

miR-221 and miR-222 are expressed by a common gene cluster on the X chromosome and share the same seed sequence (58). Our data show that these two miRNAs displayed a coexpression pattern and changed simultaneously, as they clustered together in our hierarchical clustering analysis. They were downregulated in the SCH+ATH, SCH and ATH groups, but there was no significant difference between the NC and SCH groups, as well as between SCH+ATH and ATH cases, implying that the changes may be mainly simultaneous with vascular proliferation rather than with endothelial injury. Spearman correlation analysis suggested that miR-221 and miR-222 were negatively correlated with TSH, TC, LDL-c and CIMT but positively correlated with NO and FMD. miR-221 and miR-222 are the most relevant identifiers of ATH, and their correlation with endothelial injury-related indicators (FMD and NO) is not the most prominent. We attributed these results to endothelial dysfunction as just one of the mechanisms of ATH. These results indicated that the expression of these two miRNAs may be involved in the process of endothelial injury in the middle and late stages of ATH instead of early-stage injury. NO synthesized by endothelial nitric oxide synthase protects vascular function by enhancing vasodilation and inhibiting platelet aggregation, monocyte adhesion and smooth muscle cell proliferation (59). In the process of ox-LDL-induced EC apoptosis, another study found that miR-221 and miR-222 had negative regulatory effects on the expression of the endothelial transcription factor Ets-1 and its downstream target gene p21. Overexpression of miR-221 and miR-222 could partially protect HUVECs from ox-LDL-induced apoptosis death (25).

Our data showed that miR-21-5p was significantly upregulated in patients with SCH, SCH+ATH, and ATH groups contrast with NC groups, and miR-21 in the SCH+ATH group was higher than SCH-alone and ATH-alone groups, whereas no significant differences were found between ATH and SCH groups. Spearman’s correlation analysis demonstrated miR-21 was positively correlated with TSH and negatively correlated with FMD and NO. Moreover, no significant correlations between TC, TG, or LDL-c and the expression level of miR-21 was found. Suggesting that the upregulation of miR-21 in SCH may be directly influenced by TSH rather than hyperlipidemia. The ROC curve analysis indicated that miR-21-5p had the largest AUC for detection of all patients with SCH. Suggesting that miR-21-5p may have specific expression patterns in all SCH patients including the SCH and SCH+ATH groups. miR-21 may be involved in the pivotal regulators of endothelial dysfunction processes in patients with SCH. Increased miR-21 expression is involved in circulating monocytes of mice, with aortic ATH and patients with coronary heart disease (28). Moreover, no correlation between blood parameters and the expression level of miR-21-5p was found, suggesting that the upregulation of in SCH may be directly influenced by TSH rather than hyperlipidemia. A previous study indicated that elevated TSH levels directly induced oxidative stress and damaged endothelial function, and the connection between SCH and ATH may not be entirely explained by dyslipidemia (13).

The advantages of this study include repeated measurement of thyroid function in all individuals within 6 months, avoiding the labelling of transient TSH elevation as subclinical hypothyroidism; determination of serum NO level and blood flow- mediated vasodilation function; measurement of the carotid intima to quantify endothelial function damage indicators; and detection of miRNA-based global expression level. To determine whether serum miRNA levels were confounded by the baseline characteristics, correlations between candidate miRNAs and clinical characteristics, including age, gender, and BMI, were performed. In addition, this study re-evaluated the correlation between TSH and ATH from a new perspective.

The limitations of our study is that, due to the low prevalence of SCH and ATH, we can only provide a preliminary study of a small sample of patients. Second, the number of patients lost to follow-up was greater, which does not provide a more convincing follow-up result.

## Conclusions

In summary, miR-21-5p may be involved in the atherosclerosis process in patients with subclinical hypothyroidism. miR-150-5p may be sensitive risk markers for predicting endothelial dysfunction in patients with atherosclerosis. The current study provides new insight into the significance of endothelial dysfunction-specific circulating miRNA expression patterns in patients with SCH. However, the mechanisms underlying the regulation disorders of the serum miRNA levels remain to be elucidated. Prospective large-scale studies are required to determine the potential value of circulating miRNAs as risk markers of ATH in patients with SCH.

## Data availability statement

The original contributions presented in the study are included in the article/**Supplementary Material**. Further inquiries can be directed to the corresponding authors.

## Ethics statement

The studies involving human participants were reviewed and approved by the ethics committee of the Second Affiliated Hospital of Shandong First Medical University. The patients/participants provided their written informed consent to participate in this study.

## Author contributions

Corresponding author XZ is responsible for the design and overall implementation, XY is responsible for the implementation of the experiment and paper writing, YW is responsible for case collection, LW is responsible for statistical analysis, and AC is responsible for the ultrasonic examination of samples. All authors contributed to the article and approved the submitted version.

## Acknowledgments

We are grateful to the patients who participated in this study. We also thank the Higher Educational Science and Technology Program of Shandong Province, China (Grant No. J17KA246); Shandong Provincial Key Laboratory of Endocrinology and Lipid Metabolism (Grant No. SDkeylab-Endo&LiMe2019-01); Natural Science Foundation of Shandong Province of China (Grant No. ZR2017LH023); Medical and Health Project of Shandong Province, China (Grant No. 2015WS0096) as well as Academic Promotion Program of Shandong First Medical University (Grant No. 2019QL017).

## Conflict of interest

The authors declare that the research was conducted in the absence of any commercial or financial relationships that could be construed as a potential conflict of interest.

## Publisher’s note

All claims expressed in this article are solely those of the authors and do not necessarily represent those of their affiliated organizations, or those of the publisher, the editors and the reviewers. Any product that may be evaluated in this article, or claim that may be made by its manufacturer, is not guaranteed or endorsed by the publisher.

## Abbreviations

NC, Normal control; SCH, Subclinical hypothyroidism; ATH, Atherosclerosis; FT4, Free thyroxine; FT3, Free triiodothyronine; HDL-C, High density lipoprotein-cholesterol; TC, Total cholesterol; TG, Total cholesterol; LDL-C, Low density lipoprotein-cholesterol; SBP, Systolic pressure; DP, Diastolic pressure; BMI, Body mass index; MiRNA/miR, MicroRNA; IMT/CIMT, Carotid intima-media thickness; VEGF, Vascular endothelial growth factor; TSH, Thyroid-stimulating hormone; NO, Nitric oxide; FMD, Flow-mediated dilatation.
