# Supplementary material for: Expression patterns of serum MicroRNAs related to endothelial dysfunction in patients with subclinical hypothyroidism
Source: Front Endocrinol (Lausanne). 2022 Sep 6;13:981622. doi: 10.3389/fendo.2022.981622 (PMC9485940; doi:10.3389/fendo.2022.981622)
Supplement: Supplementary file 1 [file Table_1.docx]

**Supplementary table1.** **Information of candidate human mature serum microRNAs used in this study.**

| Assay Name | miRBase Accession | Mature miRNA  Sequence |
| --- | --- | --- |
| hsa-miR-21-5p | MIMT0000076 | ucccugagacccuuuaaccuguca |
| hsa-miR-150-5p | MIMAT0000451 | cacugguacaaggguugggaga |
| hsa-miR-126-3p | MIMAT0000445 | ucguaccgugaguaauaaugcg |
| hsa-miR-210 | MIMAT0000267 | cugugcgugugacagcggcuga |
| hsa-miR-221-3p | MIMAT0000278 | agcuacauugucugcuggguuuc |
| hsa-miR-222-3p | MIMAT0000279 | agcuacaucuggcuacugggu |
